# Electrolysis for the Removal of Superfluous Hairs

**Published:** 1889-07

**Authors:** H. Montague

**Affiliations:** Detroit, Mich.


### ELECTROLYSIS FOR THE REMOVAL OF SUPERFLUOUS HAIRS.

By H. MONTAGUE, M. D., DETROIT, MICH.

In an experience extending over some ten years, I have treated upwards of fifteen hundred cases with varying success, rarely finding, though, that more than ten per cent. of the hairs return after the first series of operations.

Since the adoption of electrolysis for this purpose the operation has undergone many modifications, notably in the intensity of the current and in the fineness of the needles used.

Regarding the intensity of the current Dr. L. Brocq, of Paris, in 1866, considered as necessary a current strength of from fifteen to eighteen milliamperes; but in April, 1888, he admits that only from three to five milliamperes are necessary to obtain the best results.

In my own practice I never exceed five, and rarely employ more than three milliamperes. The outfit necessary for the removal of superfluous hairs consists of a galvanic battery, a fine needle holder and needles, epilation forceps, and above all a reliable milliamperemeter. I cannot find words strong enough in condemnation of the physician who attempts to use the galvanic current, no matter for what purpose, without having in circuit a reliable milliamperemeter. Where the Edison incandescent light wire can be made use of, the rheostat made for me by the Michigan Electrical works of this city, is far preferable to any form of battery, being cleaner, cheaper and more constant.

Almost every one practicing electrolysis has his own idea of needle holder; for my purpose I prefer a long and delicate holder, resembling an artist's brush. Jeweler's brooches, Nos. 5 and 7, form the best needles for the work, and by drawing the temper they can bend in any desired direction, thus obviating the placing of either the patient or oneself in a strained position.

I do not agree with some operators that the circuit should not be closed until the needle is inserted, as by the anodal application of a 15 per cent. solution of cocaine, I am able completely to obtund the pain resulting from the passage of the needle through the cutaneous envelop.

The length of a sitting must naturally depend upon the extent of the surface to be operated upon, the urgency of the case, and the endurance of the patient, usually in cases involving the face I limit the seance to two hours, repeated two or three times a week; in treating the arms, etc., longer, and if necessary, daily sittings can be supported. The amount of irritation created depends upon the care with which the operation is performed, and the sensitiveness of the patient's skin. I rarely find it fail to yield to hot applications of distilled extract of witch-hazel and water, one to four.

In conclusion I would say that I am convinced that in electrolysis we have not only a perfectly safe, but absolutely reliable method of permanently obliterating blemishes that have proved a source of mental anguish to many. At the same time I am not infrequently assailed with the remark, "I have tried electrolysis and it is no good." Doubtless failure is possible, but it should be attributed to the want of skill and technical knowledge on the part of the operator, and not to an operation which has been amply proved to be successful.

Regarding any scar resulting from the operation, I do not consider any such trouble possible, unless through the use of currents of too great intensity, an accident rendered impossible if a milliamperemeter is used, or through the removal of hairs in too close proximity at one sitting.—*Times and Register.*

